# Psychiatric emergency admissions during and after COVID-19 lockdown: short-term impact and long-term implications on mental health

**DOI:** 10.1186/s12888-021-03469-8

**Published:** 2021-09-24

**Authors:** Julia Ambrosetti, Laura Macheret, Aline Folliet, Alexandre Wullschleger, Andrea Amerio, Andrea Aguglia, Gianluca Serafini, Paco Prada, Stefan Kaiser, Guido Bondolfi, François Sarasin, Alessandra Costanza

**Affiliations:** 1 Department of Psychiatry and Department of Emergency, Emergency Psychiatric Unit (UAUP), Geneva University Hospitals (HUG), 1211 Geneva, Switzerland; 2 Adult Psychiatry Division, Department of Psychiatry, University Hospital of Geneva (HUG), Geneva, Switzerland; 3 Department of Neuroscience, Rehabilitation, Ophthalmology, Genetics, Maternal and Child Health (DINOGMI), Section of Psychiatry, University of Genoa, 16132 Genoa, Italy; 4 IRCCS Ospedale Policlinico San Martino, 16132 Genoa, Italy; 5 Faculty of Medicine, University of Geneva (UNIGE), 1206 Geneva, Switzerland; 6 Department of Psychiatry, Service of Liaison Psychiatry and Crisis Intervention (SPLIC), Geneva University Hospitals (HUG), 1211 Geneva, Switzerland; 7 Department of Emergency, Emergency Medicine Unit, Geneva University Hospitals (HUG), 1211 Geneva, Switzerland

**Keywords:** Coronavirus, COVID-19 pandemic, Depression, Emergency department, Public mental health, Psychiatric admissions, Psychotic episode, Substance use disorder, Suicide, Suicidal behavior

## Abstract

**Background:**

The ‘lockdown’ measures, adopted to restrict population movements in order to help curb the novel coronavirus disease 2019 (COVID-19) pandemic, contributed to a global mental health crisis. Although several studies have extensively examined the impact of lockdown measures on the psychological well-being of the general population, little is known about long-term implications. This study aimed to identify changes in psychiatric emergency department (ED) admissions between two 8-week periods: during and immediately after lifting the lockdown.

**Methods:**

Socio-demographic and clinical information on 1477 psychiatric ED consultations at the University Hospital of Geneva (HUG) were retrospectively analyzed.

**Results:**

When grouped according to admission dates, contrary to what we expected, the post-lockdown group presented with more severe clinical conditions (as measured using an urgency degree index) compared to their lockdown counterparts. Notably, after the lockdown had been lifted we observed a statistically significant increase in suicidal behavior and psychomotor agitation and a decrease in behavior disorder diagnoses. Furthermore, more migrants arrived at the HUG ED after the lockdown measures had been lifted. Logistic regression analysis identified diagnoses of suicidal behavior, behavioral disorders, psychomotor agitation, migrant status, involuntary admission, and private resident discharge as predictors of post-lockdown admissions.

**Conclusions:**

Collectively, these findings can have implications concerning the prioritization of mental health care facilities and access for patients at risk of psychopathological decompensation in time of confinement policies, but above all, provide a foundation for future studies focusing on the long-term impact of the pandemic and its associated sanitary measures on mental health.

**Trial registration:**

Research Ethics Committee of Geneva, Registration number 2020–01510, approval date: 29 June 2020.

## Background

For more than one year since the World Health Organization (WHO) declared the novel coronavirus disease 2019 (COVID-19) a pandemic, countries around the world have implemented various policies in an attempt to mitigate its spread [1, 2]. A growing body of scientific evidence has shed light on the impact of different socioeconomic and healthcare-related conditions on mental health [3–5]. For example, economic disruptions have triggered various psychological morbidities for the unemployed [6] while chaotic medical responses have fueled distress among healthcare workers [7–9]. Furthermore, global lockdown measures have had effects on psychological and emotional well-being for the general population [10, 11] as well as for vulnerable individuals (elderly, young adults, students, patients with chronic somatic disorders) [12–14], and those with a history of mental health disorders [15]. The impact of COVID-19 on the mental wellness of our society is, therefore, likely to be long-lasting and/or may become fulminant after the pandemic has subsided [16, 17].

Documenting the progression of such measures and their impact during the entire pandemic time, particularly during and post-lockdown periods, is of urgent importance to glean clinically-relevant insights that ensure optimal responses to future public health emergencies. Lockdown was first instated in Wuhan; initially faced with criticism for its harsh impact on society, this pandemic response model was subsequently implemented in many countries around the world [18]. Following the first case of SARS-CoV2 infection in Switzerland on February 25, 2020 [19], multiple clusters of infections were confirmed throughout the country [20], triggering the Federal Council to progressively adopt sanitary measures, including movement restrictions, the prohibition of large gatherings, and the mandatory closure of schools, stores, restaurants, and entertainment venues [21]. These lockdown-like measures were gradually relaxed in mid-May 2020 as the epidemiological situation improved in Switzerland.

Although several studies have examined the impact of the COVID-19 pandemic on the mental health of the general population worldwide [22, 23], little is known to date about the impact of removing lockdown measures on psychiatric admissions at the emergency departments (EDs). This information could inform on possible “rebound effects” following the imposition of lockdown measures as well as about their effects on the psychological well-being of both, general and clinical populations, because EDs are considered a frontline service that can very rapidly detect any changes [24–26].

Here, we analyze and compare the socio-demographic and clinical characteristics of patients admitted to the adult psychiatric ED of the University Hospital of Geneva (HUG) during two 8-week periods, the first coinciding with the lockdown and the second coinciding with the period immediately after the lockdown measures had been lifted. The aim of our study is to understand the possible impact of the lifting of lockdown measures on the mental health status of different patient classes.

## Methods

### Study design

A retrospective analysis of 1477 consultations at the psychiatric ED of the HUG, Geneva, Switzerland, between March 16 and July 5, 2020, was conducted. The HUG psychiatric ED is the only psychiatric ED in Geneva and forms part of the HUG general ED, which is in an urban ED. Patients can present directly to the psychiatric ED or be transferred from psychiatric/somatic outpatient facilities. All the admitted subjects were evaluated by a medical doctor and recruited without any particular inclusion/exclusion criteria. Informed consent from the patients was also waived because we argued that requesting consent would have introduced a selection bias. These consultations were divided into two subgroups based on admission date. Consultations performed from March 16 to May 10, 2020, were included in the lockdown group while those occurring between May 11 and July 5, 2020, comprised the post-lockdown group. The lockdown measures adopted in Switzerland were less severe than those implemented in other countries (private and public gatherings were prohibited, all non-essential businesses restaurants and bars were closed, but there were no strict stay-at-home orders). Given that the formal definition of lockdown differed from elsewhere, we demarcated the beginning and end of the lockdown in Switzerland by the respective dates when schools closed and reopened. Moreover, the schools reopening, as of May 11, was accompanied by the gradual reopening of non-essential stores.

All procedures in this study were carried out in agreement with the 2013 Declaration of Helsinki and reviewed and approved by the Research Ethics Committee of Geneva, Switzerland under the registration number 2020–01510 (approval date: June 29, 2020).

### Clinical assessment

The patients’ demographic and clinical information was collected according to previously published procedures [26, 27]. The parameters included were age, sex, familial/marital/residential statuses, diagnosis upon admission based on the EST® (Echelle Suisse du Tri, HUG, Switzerland), one of the two screening platforms recommended by the Swiss Society for Emergency Medicine and Rescue, ED admission modes (referral by general practitioners/psychiatrists/other HUG departments, self-admission, or by ambulance/police), admission times (8–19 h, 19–24 h, or 24–8 h; weekdays or weekends), ED stay duration (in hours), and the clinical outcomes (discharge decisions by an ED psychiatrist: involuntary/voluntary hospitalization, private resident discharge). The urgency degree of each admission was also assessed by the EST®, which includes four levels of urgency: 1 - very urgent, life-threatening condition; 2 - pathological, non-life-threatening but with the potential to rapidly deteriorate; 3 - pathological but not time-sensitive (e.g., the patient arrives in a stable condition); and 4 - medically stable and not requiring urgent care.

### Statistical analysis

Clinical data, presented as means ± standard deviations (SD) or counts and percentages, were assessed for normal distribution by the Kolmogorov-Smirnov test. Pearson’s chi-square test with Yates’ correction and a t-test for independent samples were employed for statistical comparisons. We used one variable admission period (lockdown period = 0, post lockdown period = 1). Then, we used a stepwise regression analysis with backward selection, i.e., we started by including all candidate variables and tested how the deletion of a variable affected the statistical significance of the fit. More specifically, we deleted those variables whose removal from the model resulted in the least statistically significant deterioration of the model fit and repeated this process until no further variables could be deleted. This approach was intended to eliminate potential collinearities among different predictors of patient admissions. We then used all significant variables for a univariate analysis and report only those variable that were statistically significant giving their odds ratio (OR) and confidence interval (CI) 95%. All the analyses were performed using the Statistical Package for Social Sciences version 25.0 (SPSS Inc., Chicago, IL, USA) with a statistical significance threshold of *p* < 0.05 (two-tailed). Regarding the interpretation of the specific variables, we used the same approach as in a previous study [27].

## Results

### Descriptive statistics

Overall, the HUG psychiatric ED provided 668 and 809 consultations during and after the lockdown, respectively. This represents a post-lockdown increase in consultations of 21% compared to the lockdown period. Based on a univariate analysis, we observed a statistically significant difference in the distribution of the severity of presentations based on the degree of urgency (*p* = 0.009). Notably, we observed a statistically significant post-lockdown increase in suicidal behavior (SB), (*p* = 0.029) and psychomotor agitation (*p* < 0.001) as well as an overall decrease in behavior disorder diagnoses (p < 0.001). For “suicidal behavior”, we only considered recent suicidal behavior, i.e., occurring in association with the current presentation at the psychiatric ED. From a socio-demographic point of view, the post-lockdown group contained more consultations by migrants (3.8% vs. 0.7%, *p* < 0.001). It should be noted that by “migrant” we mean a person who arrived in Switzerland from a very low-income country, often as a refugee or asylum seeker, i.e., they do not have a residence permit but only possess “migrant status” which means that they must live in special residences that are subsidized by the Swiss state. Other differences between the two subgroups are summarized in Table 1**.**

**Table 1:** Comparison of sociodemographic and clinical characteristics of patients admitted to the psychiatric emergency department (ED) during the “lockdown” and “post lockdown” period in 2020

Socio-demographic and clinical characteristics	“Lockdown” (***N*** = 668)	“post lockdown” (***N*** = 809)	Chi-squared/***t***-test	***p***-value
Female Gender, N (%)	345 (51.6)	399 (49.3)	.792	.373
Current age, year, mean ± SD	39.81 ± 16.71	40.13 ± 17.33	.358	.720
Familial status, N (%)				
Unmarried/not in relationship	387 (58.1)	453 (56.2)	.577	.902
Married/in a relationship	130 (19.5)	168 (20.8)		
Separated/divorced	129 (19.3)	159 (19.7)		
Widowed	21 (3.1)	26 (3.3)		
Residential status (where the patient comes from to be admitted to the ED), N (%)				
Private residence	530 (79.3)	645 (79.7)	18.725	<.001*
Foster home, hotel	80 (12.1)	91 (11.2)		
Homeless	53 (7.9)	42 (5.2)		
Migrants	5 (0.7)	31 (3.8)		
Referral source, N (%)				
Private psychiatrist	5 (0.7)	4 (0.5)	8.441	.208
General practitioner	13 (1.9)	28 (3.5)		
HUG	19 (2.8)	18 (2.2)		
Self-referral	249 (37.3)	305 (37.7)		
Police	75 (11.2)	68 (8.4)		
CAMSCO	2 (0.1)	4 (0.5)		
By Ambulance	305 (45.7)	382 (47.2)		
Urgency degree, according to EST®, N (%)				
Degree 1	126 (18.9)	212 (26.2)	11.474	.009*
Degree 2	303 (45.4)	336 (41.5)		
Degree 3	206 (30.8)	227 (28.1)		
Degree 4	33 (4.9)	34 (4.2)		
Diagnosis, N (%)				
Psychotic episode	31 (4.6)	31 (3.8)	49.333	<.001*
Manic/hypomanic episode	17 (2.5)	11 (1.4)		
Depression/anxiety	175 (26.1)	197 (24.4)		
Suicidal behavior	141 (21.1)	210 (26.0)		
Substance use disorder	62 (9.3)	52 (6.4)		
Behavioral disorder	126 (18.9)	86 (10.6)		
Psychomotor agitation	52 (7.8)	127 (15.7)		
Somatic problem	64 (9.7)	95 (11.7)		
Arrival hour, N (%)				
8-19 h	329 (49.3)	413 (51.1)	.742	.690
19-24 h	206 (30.8)	248 (30.7)		
24-8 h	133 (19.9)	148 (18.2)		
Arrival day, N (%)				
Weekday	465 (69.6)	578 (71.4)	.594	.441
Weekend	203 (30.4)	231 (28.6)		
Type of discharge (where the patient is referred at the time of discharge from the ED), N (%)				
Voluntary admission	107 (16.0)	90 (11.1)	11.418	.010*
Involuntary admission	86 (12.9)	130 (16.1)		
Private residence	396 (59.3)	510 (63.0)		
Others	79 (11.8)	79 (9.8)		
Duration of visit, in hours mean ± SD	6.46 ± 6.36	5.99 ± 5.51	−1.528	.127

Abbreviations: CAMSCO, Consultation Ambulatoire Mobile de Soins Communautaire; HUG, University Hospital of Geneva; * to *p* < .05. Familial status: missing data (*N* = 4)

### Logistic regression

A logistic regression analysis with ED admissions during the post-lockdown period as the independent variable generated the following ORs: 1.380 for suicidal behavior (*p* = 0.016), 2.089 for psychomotor agitation (p < 0.001), 1.636 for involuntary admission (*p* = 0.007), 4.386 for migrant status (*p* = 0.003), and 1.404 for private resident discharge (*p* = 0.009). In contrast, behavioral disorder diagnoses were significantly positively associated with the lockdown period (*p* = 0.004, OR = 0.628) (Table 2**).**

**Table 2:** Backward logistic regression analysis of the relationships between potential explanatory variables and psychiatric ED admissions in the “post-lockdown” period

	***p***-value	OR	95% CI for EXP
Suicidal behavior (SB)	.016*	1.380	1.062–1.793
Behavioral disorder	.004*	.628	.458–.862
Psychomotor agitation	<.001*	2.089	1.454–3.002
Migrant	.003*	4.386	1.680–11.449
Involuntary admission	.007*	1.636	1.145–2.339
Discharge to private residence	.009*	1.404	1.090–1.808

Abbreviations: * to p < .05

Legend: lockdown period = 0, post lockdown period = 1

## Discussion

To the best of our knowledge, this is the first report to document the post-lockdown and re-opening effects on the mental health of the clinical population during the COVID-19 pandemic in Switzerland. In a previous study conducted at the same institution, we investigated socio-demographic and clinical differences in psychiatric EDs admissions between pandemic-free and during the COVID-19 pandemic periods, using data from the same periods in 2016 and 2020, respectively [27]. We found a reduction in psychiatric ED admissions during the COVID-19 pandemic, and the admissions were positively associated with living alone and more severe psychiatric conditions (including the involuntary admissions modality). During the pandemic period, more diagnoses included suicidal behavior, psychomotor agitation, and behavioral disorders were observed [27]. Therefore, we expected that - similar to pandemic-free period - lifting the lockdown measures would result in a decrease in serious psychiatric conditions. However, the results presented in this study do not match these expectations but indicate that more severe clinical conditions (according to the EST® urgency degree) were treated post-lockdown compared to during the lockdown. We also found a significant positive association between involuntary admissions and the post-lockdown period. While research on this topic is still limited, this observed increase in more severe and involuntary admissions is consistent with our observation of more severe clinical presentations (including the diagnosis of psychomotor agitation) post-lockdown. This could be the result of a worsening mental health status in individuals with pre-existing psychiatric conditions during the lockdown due to reduced access to mental health services and the loss of social connections which would in turn lead to the observed post-lockdown increase in admissions [6, 7, 9, 14].

At the same time, alongside the increase in involuntary admissions in the post-lockdown period, which suggests more serious clinical situations, we have surprisingly observed an increase in discharges to private residences, which suggests less serious clinical conditions instead. We hypothesized that this finding was attributable, in the post-lockdown period, to the reopening of public psychiatric outpatient settings as well as the increased availability to receive patients from private psychiatrists and family physicians.

The analysis performed in this study revealed a post-lockdown increase in suicidal behavior. In other studies, increased suicidal behavior and mental health problems have been observed in different regions of the world during the lockdown period [28, 29], with some exceptions that could potentially be attributed to decreased help-seeking and hospital admission rates during these times of restricted activities [30] or other resiliency factors that protect against lockdown-induced psychiatric complications [23, 31]. McIntyre and colleagues [30] performed a longitudinal analysis of suicide rates in a cohort of 760 psychiatric ED admissions in Ireland during the early and late phases of the lockdown. They observed a sharp decrease in suicidal behavior during the early months of the lockdown, followed by a compensatory increase in suicidal behavior in the subsequent months peaking as the lockdown measures began to be removed. These findings are consistent with those reported in our study and support the hypothesis about the long-term impact of a lockdown on suicidal behavior that persists even after lockdown measures have been lifted. Several factors have been found to contribute to suicidal ideation and behavior, including, also in the long-term, the presence of economic stressors, increased consumption of addictive substances (drugs and alcohol), domestic violence, intense exposure to anecdotes of pessimism and helplessness, and persistent feelings of entrapment, isolation, and loneliness [32–34]. All of these factors can interact and have a synergistic effect, thus creating a vicious circle [35].

It should be noted that all the aforementioned studies are rather heterogeneous in that they employed different sampling approaches and methodological instruments and are therefore difficult to compare. Moreover, the situation is still evolving as were are still entering new phases of the pandemic (new lockdown and post-lockdown cycles). However, based on the most recent summary analyses, two dynamics seem to emerge: higher lockdown rates of suicidal behavior in less industrialized countries and lower lockdown rates of suicidal behavior in more industrialized countries [36, 37].

In contrast to our findings, some studies found that the lifting of the lockdown had no significant impact on mental health [38, 39]. Richter and colleagues recently reviewed the literature on mental health problems in general (and not only clinical) populations of several countries during and after the first lockdown [23]. Despite methodological inconsistencies between the included studies, they could report a slight overall decrease in mental health disorders and suicidal behavior after the first lockdown had been lifted. This result differs from our findings and may be due to the fact that the studies included in their review employed different sampling approaches and methodological instruments (e.g., self-reporting and online surveys vs. hospital admissions). Also, socio-economic differences, health policy-related issues, and cultural factors of resilience (e.g., social support, education level and psychological flexibility) may also affect an individual’s susceptibility to the negative impacts of a lockdown [23, 35]. Furthermore, while we only included a very narrow part of the general population, namely individuals admitted to a psychiatric emergency department, the results by Richter and colleagues are based on studies that included a much broader spectrum of the general population.

From a socio-demographic viewpoint, our study revealed a significant increase in the number of migrants being admitted to psychiatric EDs in the post-lockdown period. This phenomenon might be attributed to the accumulating healthcare burden in this demographic group throughout the pandemic, particularly during the lockdown period, where the impact has been demonstrably prominent. For example, studies focusing on migrants reported an increase in mental health disorders in this demographic [40, 41]. Sanitary measures associated with the lockdown have brought the lives of many migrants to a standstill as they faced increased precariousness, financial constraints, and stigmatization by the non-migrant community. Furthermore, in some migrants’ communities, preventative social distancing measures could not be implemented due to a lack of living space, which lead to numerous infection outbreaks. Thus, migrants are more susceptible to the psychological and emotional trauma of the COVID-19 pandemic. These observations might explain the influx of patients with migration background to our psychiatric ED during the post-lockdown period, i.e., once mental health access began to normalize. However, increased psychiatric ED admissions of migrants during the post-lockdown period cannot be explained by mental health or psychological issues alone and additional studies are required to elucidate the underlying associations.

Collectively, these findings emphasize the urgent need to provide access to mental healthcare during lockdowns, particularly for those who are more likely to suffer from psychiatric complications to prevent their manifestation during the post-lockdown period. Moreover, these findings suggest that additional attention should be paid to psychiatric conditions associated with involuntary admissions, severe clinical presentations, and suicidal behavior at psychiatric EDs during post-lockdown periods so as to be properly prepared for such cases.

These objectives could be achieved by various means, including increasing the mobility of psychiatrists and nurses for in-home care, equipping psychiatric ED with high isolation standards against infections, and the use of telepsychiatry, defined as “the delivery of mental health care in the form of live and interactive videoconferencing” [26, 42]. In the context of telepsychiatry, patients could be evaluated and advised for treatment remotely. Telepsychiatry could provide uninterrupted care for psychiatric patients in real-time to help them cope with mild psychiatric issues during lockdowns so these problems will not transform into more severe clinical presentations in the long term (i.e., post-lockdown period). Furthermore, telepsychiatry could be considered a remote support mechanism to generate a sense of interconnectedness for patients who are suffering from loneliness, hopelessness, and helplessness, which have been significantly associated with increased suicidal behavior risk [43, 44]. Of particular relevance to psychiatric EDs [45], remote services can be implemented by various means, and in an unexpectedly fortuitous manner, the COVID-19 pandemic has created an opportunity to utilize this technology to improve mental health access, care quality, and immediacy for psychiatric patients.

### Limitations

This study needs to be interpreted in the light of several limitations. First, given its being retrospective and single-center, the findings need to be validated in a more diverse patient population before they can be generalized to other contexts. Secondly, two relatively brief periods of 8 weeks (for a total of 16 weeks) were compared and it is difficult to precisely delineate the differential impact of acute vs chronic lockdown and/or reopening on the frequencies of psychiatric ED admissions and their characteristics. Third, unlike many other countries with severe lockdowns, sanitary measures in Switzerland are more flexible with the absence of strict stay-at-home orders; these variable lockdown protocols might differentially impact the clinical features of psychiatric ED admissions, which would limit the representability of our findings. Fourth, these findings are associated with the initial lockdown wave and COVID-19 infections and, therefore, they only provide limited insight into the long-term impact of multiple waves of infections and lockdowns on mental health. Finally, a more systematic analysis that goes beyond the sample size of the current study as well as those previously published and psychiatric ED admissions during various phases of the pandemic is warranted.

## Conclusions

The observations described in this study have important pragmatic implications concerning the organization of psychiatric ED personnel to best accommodate these clinical and epidemiological trends. Our findings also provide a foundation for further research to elucidate the long-term consequences of lockdown on mental health. The right to benefit from mental health services [46], even during a lockdown, seems to emerge as a pivotal factor for avoiding the “rebound” effect during the post-lockdown period. We believe that future challenges and perspectives should include research on both, general and clinical population, with a larger number of patients and a longer observation period.

## Data Availability

The datasets used and/or analyzed during the current study are publicly not available due to limitations of ethical approval involving the patient data and anonymity but are available from the corresponding author on reasonable request.

## Abbreviations

CI: confidence interval
COVID-19: Coronavirus disease 19
ED: Emergency department
EST: Echelle Suisse de Tri
HUG: University Hospital of Geneva
OR: Odds ratio
SB: Suicidal behavior
SD: Standard deviation
SPSS: Statistical Package of Social Sciences
WHO: World Health Organization
